# A Modeling-Derived Hypothesis on Chronicity in Respiratory Diseases: Desensitized Pathogen Recognition Secondary to Hyperactive IRAK/TRAF6 Signaling

**DOI:** 10.1371/journal.pone.0005332

**Published:** 2009-04-24

**Authors:** Tingting Zhang, Kyung W. Song, Mohammad Hekmat-Nejad, David G. Morris, Brian R. Wong

**Affiliations:** Roche Palo Alto LLC, Palo Alto, California, United States of America; Johns Hopkins School of Medicine, United States of America

## Abstract

Several chronic respiratory diseases exhibit hyperactive immune responses in the lung: abundant inflammatory mediators; infiltrating neutrophils, macrophages, lymphocytes and other immune cells; and increased level of proteases. Such diseases include cystic fibrosis (CF), chronic obstructive pulmonary disease (COPD) and severe/neutrophilic asthma. Paradoxically, patients with these diseases are also susceptible to detrimental bacterial infection and colonization. In this paper, we seek to explain how a positive feedback mechanism via IL-8 could lead to desensitization of epithelial cells to pathogen recognition thus perpetuating bacterial colonization and chronic disease states in the lung. Such insight was obtained from mathematical modeling of the IRAK/TRAF6 signaling module, and is consistent with existing clinical evidence. The potential implications for targeted treatment regimes for these persistent respiratory diseases are explored.

## Introduction

Human epithelial cells are the first line of defense against external pathogens and particulates. The innate immune responses mediated by these cells are important for host defense against bacteria and viruses [1], [2]. A number of TIR-domain containing receptors play important roles in the initial pathogen recognition by Toll-Like Receptors (TLRs) or the triggering of adaptive responses via cytokine receptors (e.g. receptors for IL-1 or IL-18). Signaling through the downstream MyD88-IRAK1/4-TRAF6 cascade has been shown to be essential for biological events mediated by Toll-Like Receptors against some bacteria (TLR2, TLR5), single strand RNA (TLR7) or DNA (TLR9) viruses. These signals will be referred as extrinsic stimuli in this paper.

**Figure 1:**
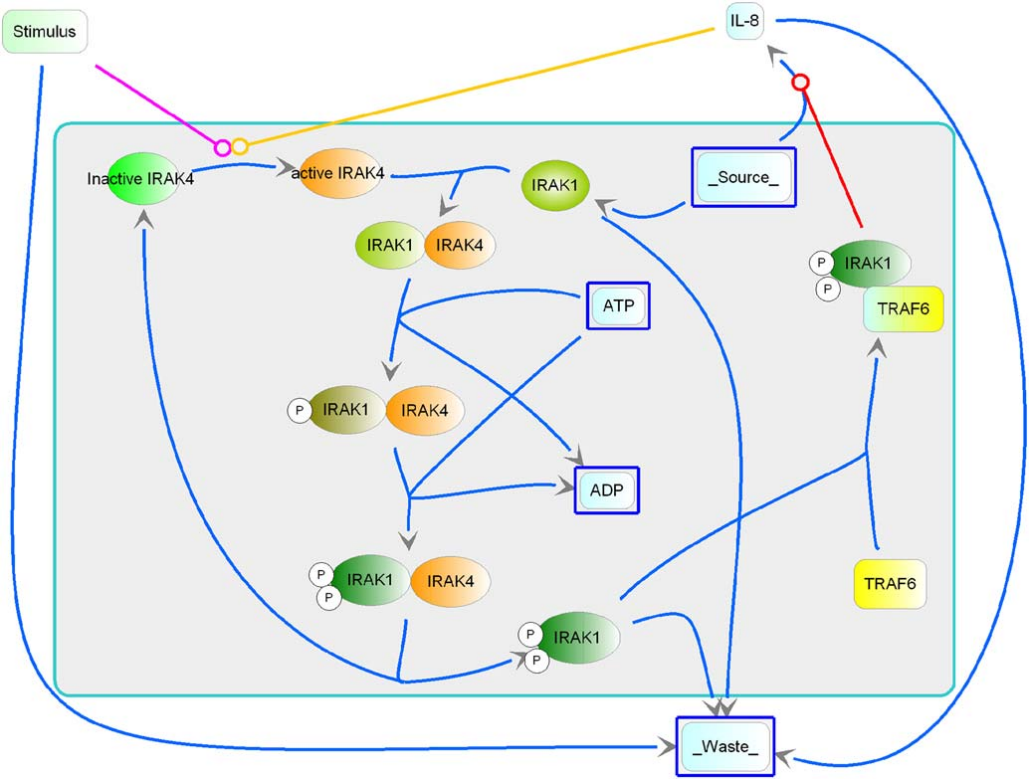
A schematic view of the IRAK/TRAF6 signaling module. Recruited by activated TIR containing receptors (for stimuli like cytokines, bacteria, or viruses, pink arrow with a closed-circle arrowhead) through interaction with MyD88, the IRAK4/IRAK1 complex undergoes multi-step phosphorylation to produce activated IRAK1 proteins. Activated IRAK1 proteins complex with TRAF6 to induce production of IL8 (red arrow), which forms a positively feedback loop to activate IRAK4 (dark yellow arrow). The activated IRAK1 protein is controlled by rapid degradation.

The IRAK4/IRAK1 complex is recruited to activated receptors by stimuli such as cytokines, bacteria, or viruses through the interaction of IRAK4 with MyD88 and additional partners. It is phosphorylated at multiple residues to produce activated IRAK1 proteins. The activated IRAK1 then forms a complex with TRAF6 to activate downstream MAPK and NF-kappaB cascades and subsequently induce the production of chemokines (for example IL-8), cytokines and antibacterial peptides. The level of activated IRAK1 proteins is controlled by the rapid ubiquitin-targeted degradation of phosphorylated IRAK1. This key signaling component in innate immunity will be referred as the core IRAK/TRAF6 module in this paper (Figure 1).

IL-8, a major chemoattractant for neutrophil recruitment, is produced by epithelial cells upon IRAK/TRAF6 activation. A positive feedback loop from IL-8 signals to NF-kappaB though the IRAK module has been reported [3], and neutrophil elastase produced by recruited neutrophils could potentially induce epithelial IL-8 production through IRAK signaling [4]. For example, recent work from Hartl et al suggested that proteases, such as neutrophil elastases, could cleave IL-8 receptor CXCR1 into fragments to activate TLR2 and its downstream IRAK signaling in epithelial cells [5]. In this paper, we refer to these mechanisms as “intrinsic stimuli”. The focus of our study was to examine how an IL-8 mediated positive feedback loop through the IRAK/TRAF6 module, induced by intrinsic or extrinsic signals, could impact the host's innate immune response in the context of localized inter-cellular events between epithelial cells and neutrophils.

A detailed schematic model of the TLR and IL-1 signaling cascades is available and the robustness of the IRAK signaling has been discussed previously [6]. This earlier model by Kitano et al focused principally on cytokine signaling feedback, systematic leukocyte development, and benevolent bacteria fauna. In our study, we focused more exclusively on epithelial cells and the effects of positive chemokine feedback on the innate immune response of these cells. We have explored how the fragilities in epithelial innate immune responses can lead to chronic disease states in lung epithelium with desensitized microbial recognition and bacterial colonization in the presence of neutrophils and inflammatory mediators.

## Results

Intrigued by the reported IL-8 positive feedback loop to IRAK signaling by Monna, et al [3], we were interested in modeling the impact of this finding on the dynamics and different steady states of the innate immune responses mediated by the IRAK/TRAF6 module. A model for the module was constructed in JDesigner [7] (graphic representation shown in Figure 1) in SBML (Systems Biology Markup Language). The model consists mainly of the core IRAK(IRAK1 and IRAK4)/TRAF6 regulatory module, with various inputs to activate IRAK4 from either the extrinsic stimulus (Figure 1, pink arrow with a circular arrowhead, a kinetic approximation of multiple signaling steps from ligand-receptor activation, MyD88 binding, to IRAK4 recruitment) or the intrinsic IL-8 feedback (Figure 1, dark yellow arrow, an approximation of activation steps of IL-8 induced neutrophil recruitment, neutrophil elastase production and subsequent feedback to the recruitment of IRAK4 by activated receptors). Activated IRAK4 then binds to IRAK1 to form a complex (IRAK1/IRAK4). Since the multi-step processes of phosphorylation of IRAK1 are mediated by the kinase activities in this complex, the reactions were modeled as Michaelis-Menten reactions using ATP as the only substrate. The IRAK1/TRAF6 complex-formation step is closely coupled with rapid ubiquitin-mediated IRAK1 degradation [8] that regulates the level of downstream output. The activated IRAK1/TRAF6 then induces IL-8 production (Figure 1, red arrow, a highly approximated estimate from the multiple-step activation through the MAPK and IKK-NF-kappaB cascades, transcription, transcript stability, translation and post-translational processing).

**Figure 2:**
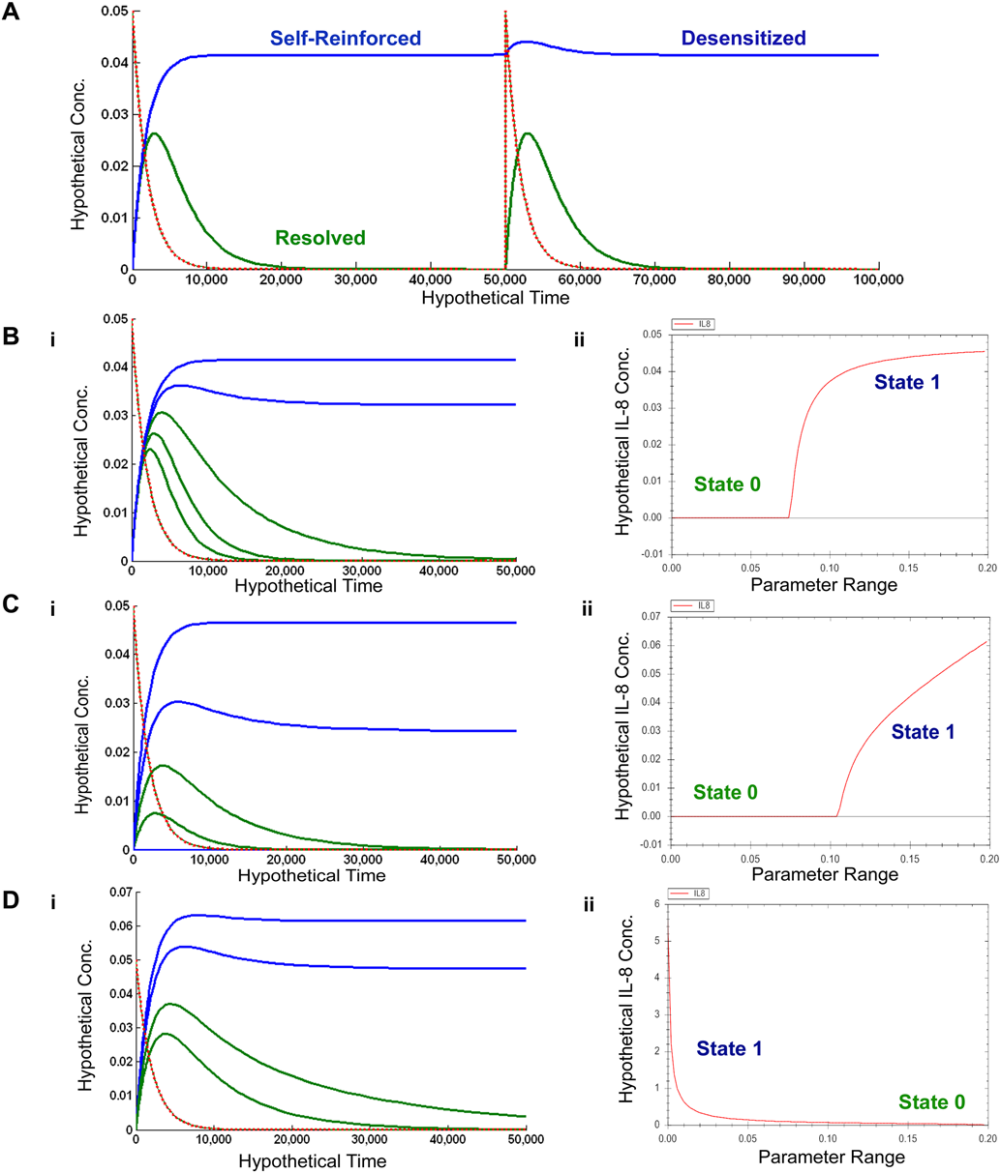
IRAK/TRAF6 response to external stimulus is dependent on the initial condition. A. Given an external stimulus (dashed red line, introduced at time 0 and decay over time), the signaling through IRAK/TRAF6 can either “resolve” (green line, representing an response of IL-8 that returns to 0) or become “self-reinforced” (blue line, representing a constitutive IL-8 level that positively feeds back to the IRAK/TRAF6 module). If a new external stimulus is introduced (dashed red line, introduced at time 50,000) to the system in a “resolved” state, a robust response of IL-8 is produced. If the system is in a “self-reinforced” state, the response to the new stimulus is “desensitized” as a much diminished response (blue line). The system states associated with “resolved” responses are defined as “State 0” (green in all panels); and those states associated with “self-reinforced” response are called “State 1” (blue). B–D: Parameter scans for the sensitive parameters whose change can introduce a switch between “State 0” and “State 1”. B. Rate of IL-8 mediated activation of IRAK4 (dark yellow arrow in Figure 1); C. IRAK1/TRAF6 induced production of IL-8 (red arrow in Figure 1); D. Disassociation rate of the IRAK1/TRAF6 complex. B(i)–D(i): Matlab time responses for various parameter values in “State 0” (green lines) or “State 1” (blue lines). B(ii)–D(ii): Parameter ranges predicted by SBW that would exhibit state changes in the final IL-8 values.

Depending on the initial cellular states at the time of stimulation, this signaling module would respond to IRAK4 activation by external stimuli (cytokines, bacteria, or viruses) with different outcomes in time series analysis. Given a pulse-like stimulus from baseline (at 0), the cells can either induce a response in IL-8 level that returns to the initial state of 0 (Figure 2A, green line, “resolved”), or reach a steady state of constitutive IL-8 expression even after the original stimuli dissipates (Figure 2A, blue line, “self-reinforced”). In subsequent discussions, we will refer to the initial cellular states that give the “resolved” responses as State 0, and those that give the “self-reinforced” responses will be referred as State 1.

The parameters that impact the state switches between “resolved” (State 0) and “self-reinforced” (State 1) of final IL-8 levels are examined through both sensitivity analyses (data not shown) and parameter scans (Figure 2B–D) using Matlab Simbiology or Systems Biology Workbench. From all the parameters in the model, sensitive parameters identified include: the rate of IL-8 mediated activation of IRAK4 (Figure 2B, dark yellow arrow in Figure 1), the rate of IRAK1/TRAF6 induced IL-8 production mediated by the processes of downstream MAPK and/or NF-kappaB signaling (Figure 2C, red arrow in Figure 1), and the dissociation rate for the IRAK1/TRAF6 complex (Figure 2D). At sensitive ranges (Figure 2Bii, 2Cii, 2Dii), small changes of these parameters in State 0 (green lines, Figure 2Bi–2Di) can push the system to fall into the constitutive IL-8 producing State 1 (blue lines, Figure 2Bi–2Di), or vice versa.

There are many possible intracellular or extracellular factors that could impact these parameters to create a heightened innate immune response: viral/bacterial infections [9], genetic variation in proteases/anti-protease or receptor activities [10], [11], other cytokines that raise innate immune baseline responses, or genetic variation or intracellular modulation of IL-8 production [12]–[14]. Cellular states of heightened innate immunity during the time of stimulation can condition cells preferentially to State 1 leading to self-enforcing IL-8 production and release, high recruitment of neutrophils, and a high level of neutrophil elastase production.

An interesting result arose when we introduced a spike of external stimulus as an input for the IRAK/TRAF6 module at State 1 (Figure 2A, time = 50000). Because the host's constitutively active IL-8 could saturate the IRAK/TRAF6 signaling, the host epithelial cells could then become desensitized in responding external stimuli (Figure 2A, blue line, time > 50000). Even though the cells have fully active IRAK1/TRAF6 signaling, the host epithelial cells may, paradoxically, be effectively inert to some pathogens. This would occur particularly in situations where the IRAK/TRAF6 signaling is the only way the host can detect such pathogens and mount an effective innate immune response against them. This state of heightened cellular activation with desensitized defense predicted by this model may seem counter-intuitive at first; however, there is abundant evidence in the literature that these states exist in many inflammatory epithelial diseases, particularly in the lung.

**Figure 3:**
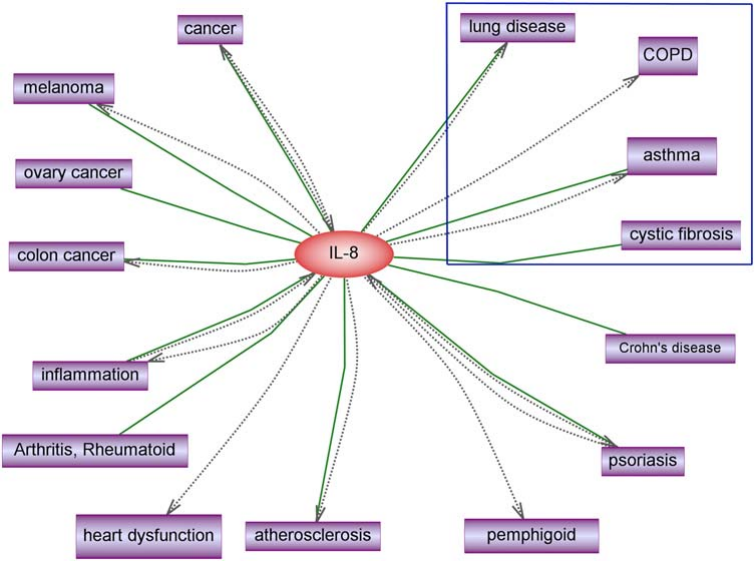
Diseases associated with IL-8 constitutive expression. A number of chronic respiratory diseases, such as cystic fibrosis, COPD and asthma (in the blue box) are identified to be associated with constitutively high level of IL-8 through text-mining PubMed abstracts in MedScan. These chronic respiratory diseases also exhibit a phenotype of susceptibility to bacteria infection. IL-8 constitutive expression is also associated with a number of cancers, and additional inflammatory diseases.

To identify the epithelial diseases with constitutive levels of IL-8, we utilized the MedScan text mining capability available in Ariadne PathwayStudio. Using IL-8 constitutive expression as the Pubmed search criteria, the top 10,000 resulting abstracts were mined for diseases that shared relationships with IL-8 (Figure 3). A number of refractory respiratory diseases were identified (blue box). Interestingly, most of these diseases that display a signature of IL-8 constitutive expression also exhibit a phenotype of susceptibility to infection by bacteria: cystic fibrosis (CF, caused by genetic mutations in CFTR-cystic fibrosis transmembrane conductance regulator), chronic obstructive pulmonary disease (COPD), neutrophilic asthma [15], and other lung diseases.

Cystic fibrosis patients have constitutive high levels of IL-8 in their blood and sputum, and their lungs are obstructed with dead neutrophils and colonized by *S. aureus* or *P. aeruginosa* [16]. Additionally, the rate of IL-8 production in response to stimulation in naïve CF cells is much faster than in healthy cells [17], [18]. Supporting our prediction (Figure 2A), CF nasal polyps exhibited constitutively higher basal, level of IL-8 and higher activity of NF-kappaB. Incubation with *P. aeruginosa* or IL-1beta resulted in less increase of IL-8 secretion by CF cells (1.4 fold) than by non-CF cells (6.3 fold, reaching the basal IL-8 level in CF cells). This is consistent with the observation that activity of NF-kappaB (contributing to the rate of IRAK/TRAF6 induced IL-8 in our model, red arrow in Figure 1) was higher at basal level in CF cells than in non-CF cells [19]. Recently, functional CFTR is reported to be a negative regulator of NF-kappaB mediated signaling [20].

### COPD

IL-8 induced neutrophil enrichment is associated with chronic obstructive pulmonary disease (COPD). Higher rates of production of IL-8 were observed in cells from smokers with COPD, and persistent inflammation is associated with COPD long after smoke cessation. [14], [21], [22]. COPD patients are also prone to exacerbations associated with bacteria infection and colonization [23]. The spectrum of the pathogens tends to overlap with those of severe asthma and CF [24].

### Neutrophilic Asthma

A higher number of severe asthma patients, compared with mild asthma patients, exhibit a disease phenotype of persistent high IL-8 level, higher neutrophil counts, and higher bacteria endotoxins in their induced sputum [15], [25]. Such an asthma subtype can be defined as neutrophilic asthma. IL-8 level has been identified in several studies as being closely associated with low lung function in asthmatic patients [26].

In summary, these disparate chronic respiratory diseases have a common theme in their disease manifestation: increased IL-8 levels, increase neutrophils, and ineffective host defense to certain pathogens. These observations suggest this model is operative in these diseases.

## Discussion

To mount a defense against pathogens or respond to other damaging or noxious stimuli, the hosts' innate immune systems need to achieve allostases; in other words, maintain sufficient innate response yet not become hyper-responsive or desensitized to microbial recognition. For the species to survive these pathological or physiological insults, there is substantial evolutionary and population diversity for these sensitive parameters in and around the IRAK/TRAF6 module. Our model predicts that the qualitative diversity in these parameters affect the host's responsiveness towards environmental pathogens [11], [27], [28]. At the extreme, these normal population variances could also contribute to propensity for the development of chronic inflammatory and infectious diseases.

**Figure 4:**
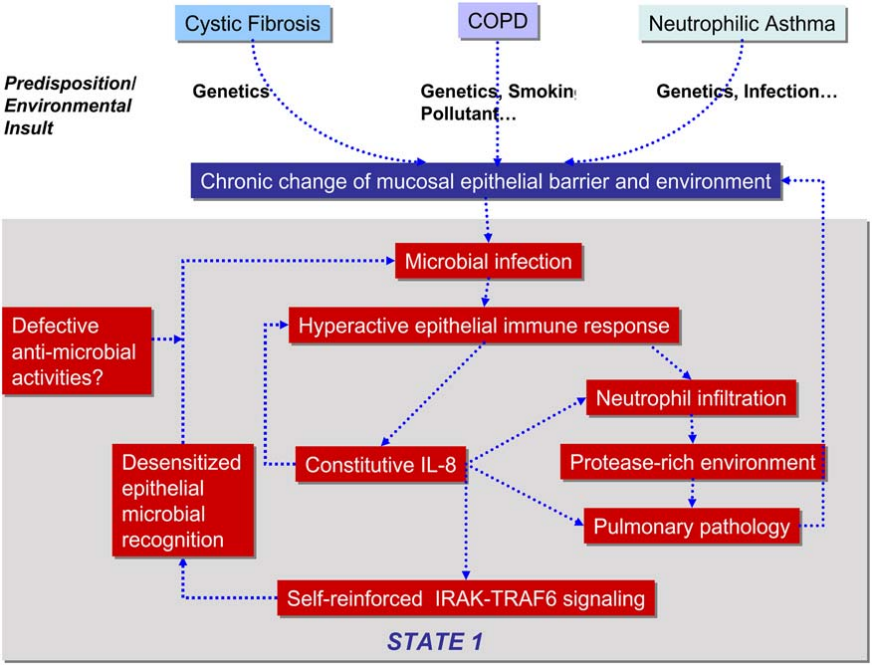
Potential mechanisms of hyper-inflammation and microbial colonization in chronic respiratory diseases. Genetic predispositions and environmental insults lead to chronic modification in pulmonary mucosal epithelial barriers in cystic fibrosis, COPD and neutrophilic asthma patients. Initial microbial infection could lead to a switch into State 1 with hyperactive epithelial innate immune responses resulting in constitutive IL-8 level, high neutrophil count, high protease levels and additional changes in pulmonary structure. Due to the intrinsic IL-8 positive feedback, bacteria recognition through TLR-IRAK-TRAF6 signaling could become desensitized. These bacteria could evade innate immunity and colonize the lung when the host's anti-microbial activities are compromised.

Genetic variation in signaling intermediates, frequency or severity of bacterial/viral infections, exposure to tobacco smoke, aging, or the presence or absence of alternative cytokine profiles are all possible susceptibility factors that can contribute to the changes in the identified sensitive parameters in the IRAK/TRAF6 signaling module. In cystic fibrosis, COPD and neutrophilic asthma, chronic inflammation and damage to the mucosal epithelial barrier could predispose patients to bacteria infection (Figure 4). The first wave of bacteria infection could trigger an epithelial immune response resulting in constitutive levels of IL-8 (State 1). High levels of IL-8 would increase recruitment of neutrophils which would secrete high levels of neutrophil proteases. Such hyperactive immune responses would lead to higher level of proteases in the epithelial environment. Higher level of proteases will then break down pulmonary structures as, for example, in COPD. IL-8 can also promote angiogenesis [29], leading to additional pathological changes in the lung.

Paradoxically, our model also predicts that constitutively expressed IL-8 could desensitize the TLR-IRAK-TRAF6 signaling. Innate immune responses to *Staphylococcus aureus* signal though IRAK-dependent TLR2 [30], [31], and *Pseudomonas aeruginosa* is recognized through IRAK-dependent TLR5 signaling [32]. In susceptible patients (State 1) at the time of infection, the host may not be able to recognize these bacteria through the respective TLRs because the downstream IRAK/TRAF6 module has been overwhelmed by intrinsic signals. When desensitized recognition is coupled with defective clearance, the pathogen can then effectively evades host immunity to colonize the host. Interestingly, the chronic respiratory diseases with constitutive IL-8 levels are prone to colonization by a similar spectrum of bacteria (including *S. aureus* and *P. aeruginosa*) as are IRAK4-deficient children with the non-responsive IRAK/TRAF6 signaling [31]. Once trapped in this state, our model predicts that cells may be slow or unable to return to normal because these bacteria are also capable of directly activating the IRAK/TRAF6 module thus triggering vicious cycles of infection, ineffective bacteria clearance, and chronic inflammation.

Our exploration of the IRAK/TRAF6 signaling module has been focused primarily on specific cell types, specifically epithelial cells and recruited neutrophils. However, there is obviously substantially more complexity in the extracellular signals and cell types that affect the epithelial environment, including monocytes, dendritic cells, mast cells, macrophages, and various lymphocytes [1], [33]. A potential weakness of the model is that it does not capture antibacterial peptide responses which are also regulated by the IRAK/TRAF6 module. Moreover, this model also does not address the potential defects in neutrophil activity in a protease-rich environment, for example, through the cleavage of chemokine receptor CXCR1 [5]. As additional signaling modules and cellular responses are defined, future disease models will need to incorporate them to better understand the fine-tuning of the signaling pathways of host immune response. Many of these chronic diseases have periods of exacerbation followed by resolution, suggesting that other feedback mechanisms in the system are important. Such counter-regulatory responses are typically robust enough to successfully return the system to the normal state. Occasionally, the detrimental combinatorial effects of these internal states and external factors in the host could override these checks and balances, resulting in chronic inflammation and bacterial colonization. In epithelial diseases where neutrophils play a major role, the IL-8 feedback and the IRAK/TRAF6 signaling module appears to be a critical component of this process.

The underlying mechanisms of the aforementioned diseases suggest that their treatment should be distinguished from diseases with similar symptoms but with different mechanisms. In chronic respiratory diseases characterized by both chronic neutrophilic inflammation and bacteria infection, this model suggests that epithelial cells may be trapped in a self-reinforcing positive feedback loop. Endogenous counter-regulatory pathways could be insufficient to resolve pro-inflammatory signaling. There would therefore be a need for external therapeutic interventions that break such a cycle. For example, Hartl et al [5] showed that inhalation of alpha1-antitripsin, a neutrophil elastase inhibitor, could restore the killing of *P. aeruginosa* in cystic fibrosis patients. Such a result can be explained by this model. Blocking neutrophil elastase would reduce the feedback from IL-8 to the IRAK/TRAF6 module by reducing the protease cleaved CXCR1 fragments, which signal to epithelial TLR2 to activate the IRAK/TRAF6 module. This model also helps to explain why impaired bacterial killing only impacts the clearance of certain bacteria in cystic fibrosis. In addition to topical treatments, our model also predicts that well-dosed systemic administration of drugs that reduce chemokine production (and are often considered to be at risk of causing increased infection in normal systems), could increase bacteria clearance and rescue from these particular disease states. Given the broad range of inflammatory diseases potentially associated with this mechanism, these results have important therapeutic implications in the treatment of chronic respiratory diseases including cystic fibrosis, COPD and neutrophilic asthma.

## Materials and Methods

### Model Construction

The IRAK/TRAF6 model was constructed in SBML (Model S1) using JDesigner2.0 in the SBW (Systems Biology Workbench www.sbml.org) package. The initial concentrations of the model species were mostly estimated. The initial parameters were either estimated or constrained using literature or experimental data [34]. The best parameterized part is the core IRAK1/ IRAK4 enzyme reactions in which the kcat is defined in s^−1^ and the Km defined in µM^−1^. The state presented in the attached model is prone to the switch between State 0 and State 1.

### Time Series Analysis, Sensitivity Analysis and Parameter Scan

Time series analyses of the model were performed in either SBW JDesigner or Matlab Simbiology. Sensitivities of parameters in regards to IL-8 level (t = 15,000) in the systems were identified using the MatLab SimBiology module with the Sundial solver. Parameter scans on all the individual parameters were performed using the Jarnac simulation service in the SBW package. Those sensitive parameters that exhibited a change from/to 0 in IL-8 levels in the scanned ranges were then simulated close to the switching range in the Matlab Simbiology parameter scan. In addition to the parameters identified in Figure 2B–2D, changes in the rate of IL-8 degradation can also switch the attached model between State 0 and State 1.

### Text Mining

Text mining of diseases associated with IL-8 constitutive levels or expression were performed using MedScan 1.0 (available from Ariadne 5.0) looking for either co-occurance or Ariadne-defined relationships. Only those diseases with at least 2 relevant references associated with IL-8 were selected. The published network was generated based on the first 10000 PubMed abstracts downloaded with the search terms “Interleukin-8” AND (“constitutive” OR “level” OR “expression”) on September 18^th^, 2008.

## Supporting Information

Model S1 The IRAK/TRAF6 model in the SBML format. Quantitative (internal and external) enzyme kinetics data and literature knowledge on structure of the signaling pathways were incorporated to construct a parameterized IRAK/TRAF6 model [34] (Hekmad-Nejad, manuscript in preparation; Zhang et al, proceeding I04 at ICSB 2007). The core IRAK enzymatic reactions were the best characterized part of the model, with other parts estimated based on constraints from the literature. The parameter sets were rested close to the state switch between State 0 and State 1.
(0.01 MB ZIP)
